# Asthma Prevention and Management for Aboriginal People: Lessons From Mi’kmaq Communities, Unama’ki, Canada, 2012

**DOI:** 10.5888/pcd13.150244

**Published:** 2016-01-14

**Authors:** Heather Castleden, Robert Watson, Tui’kn Partnership, Ella Bennett, Jeffrey Masuda, Malcolm King, Miriam Stewart

**Affiliations:** Author Affiliations: Heather Castleden, Jeffrey Masuda, Queens University, Kingston, Canada; Robert Watson, University of Calgary, Calgary, Canada; Tui’kn Partnership [Elaine Allison, Darlene Anganis, Jennifer MacDonald, Sharon Rudderham, and Laurie Touesnard, Health Directors of the 5 Mi’kmaq communities in Unama’ki (Cape Breton), Nova Scotia, Canada]; Ella Bennett, Dalhousie University, Halifax, Canada; Malcolm King, Simon Fraser University, Burnaby, British Columbia; Miriam Stewart, University of Alberta, Edmonton, Canada.

## Abstract

**Background:**

Asthma affects at least 10% of Aboriginal children (aged 11 or younger) in Canada, making it the second most common chronic disease suffered by this demographic group; yet asthma support strategies specific to Aboriginal peoples have only begun to be identified.

**Community Context:**

This research builds on earlier phases of a recent study focused on identifying the support needs and intervention preferences of Aboriginal children with asthma and their parents or caregivers. Here, we seek to identify the implications of our initial findings for asthma programs, policies, and practices in an Aboriginal context and to determine strategies for implementing prevention programs in Aboriginal communities.

**Methods:**

Five focus groups were conducted with 22 recruited community health care professionals and school personnel in 5 Mi’kmaq communities in Unama’ki (Cape Breton), Nova Scotia, Canada, through a community-based participatory research design. Each focus group was first introduced to findings from a local “social support for asthma” intervention, and then the groups explored issues associated with implementing social support from their respective professional positions.

**Outcome:**

Thematic analysis revealed 3 key areas of opportunity and challenges for implementing asthma prevention and management initiatives in Mi’kmaq communities in terms of 1) professional awareness, 2) local school issues, and 3) community health centers.

**Interpretation:**

Culturally relevant support initiatives are feasible and effective community-driven ways of improving asthma support in Mi’kmaq communities; however, ongoing assistance from the local leadership (ie, chief and council), community health directors, and school administrators, in addition to partnerships with respiratory health service organizations, is needed.

## Background

Gross health disparities are found among Canada’s Aboriginal (First Nations, Inuit, and Métis) peoples relative to that of Canadians generally (1). Colonialism and racism toward Aboriginal peoples — evident through their marginalization within social, political, economic, and health systems — have impeded access to important social determinants of health such as adequate housing, income, education, and health care, which impair the ability to achieve good health outcomes (2).

Social determinants of health are generally understood as the underlying economic and social conditions that impact health across the life course; they are the living conditions, vulnerabilities, and capacities that shape our health (3). Loppie Reading and Wien discuss the state of Aboriginal peoples’ health across proximal (eg, poverty), intermediate (eg, health care, language), and distal (eg, colonialism, racism) scales, all of which link to existing Aboriginal health inequities (2).

An important corollary is the field of health promotion. Health promotion aims to prevent ill health by focusing on the need to change sociopolitical, economic, and environmental conditions (4). Because colonization and racism are at the core of the health challenges facing Aboriginal peoples, health promotion strategies that acknowledge and apply Aboriginal ways of improving health are needed (4). This article presents research that focuses on asthma as a particular health challenge facing Aboriginal people in Canada, and it offers ways forward for culturally appropriate, community-led support for Aboriginal children with asthma and their families. Although the focus of our research is geographically specific, the health status of the world’s approximately 370 million Indigenous peoples can be described in much the same way: unjust (5); the underlying causes of their health inequities are similarly attributed, in large part, to “colonisation, globalisation, migration, loss of language and culture, and disconnection from the land” (6).

## Community Context

Although the prevalence of asthma among Aboriginal children is similar to that of all Canadian children, far fewer Aboriginal children receive treatment (7). Asthma affects more than 10% of Aboriginal children (aged 11 or younger) (8), and although the percentage of those with asthma who receive treatment has increased from 57% to 69% (9), asthma among Aboriginal peoples in general is thought to be greater than reported because of difficulties with surveying this population and the population’s lack of access to appropriate health services (eg, spirometry testing) (9). The incidence and severity of asthma and asthma symptoms in Aboriginal communities is linked to inadequate living conditions, including overcrowded housing, poor ventilation, exposure to mold and indoor tobacco smoke (10), cultural practices including burning ceremonial tobacco or other sacred medicines for smudging or cleansing, and the use of wood-burning stoves to reduce heating costs (11), lack of financial resources (2), and stressful home environments (12) that pervade many Aboriginal communities. Aboriginal peoples are also experiencing not only disproportionately higher emergency department visits but also more repeated visits than their non-Aboriginal counterparts (13). Unfortunately, with notable exceptions (14,15), research addressing social support needs for Aboriginal families living with asthma is scarce.

Research involving Aboriginal peoples has a history of neglecting the health priorities of the community members themselves (16). In response, community-based participatory research is an approach that privileges Aboriginal peoples’ perspectives and guidance in research (17). We undertook this approach in partnership with Aboriginal communities in 3 Canadian provinces: Alberta (west), Manitoba (central), and Nova Scotia (east). Each provincial site had a lead academic researcher who sought guidance from a local Aboriginal community advisory committee in terms of the intervention designs and data collection processes. Briefly, in Alberta (led by M.S.) methods included telehealth, an asthma camp (an overnight camp that provides educational and support activities through typical camp experiences of games, crafts, physical and cultural activities in order to teach children to take control and manage their asthma), face-to-face peer support groups, and a final small symposium. In Manitoba (led by J.M.), the methods included a community-based face-to-face peer support group and a large regional symposium of health care professionals and multiple community representatives. The research reported in this article is specific to the Nova Scotia site (led by H.C.), which involved 5 partnering Mi’kmaq (Aboriginal) communities (Figure) over 3 project phases. Given the uniqueness of the community-based participatory research approaches at each site, site-specific findings are reported elsewhere (for a multisite lay report, see http://www.heclab.com/wp-content/uploads/2015/02/Asthma-Study-Multi-Site-Lay-Report-Feb-2015-amended.pdf).

**Figure:**
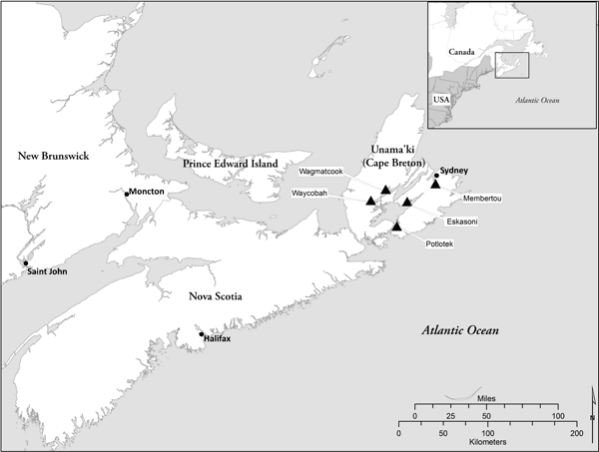
Figure. Map of Unama’ki (Cape Breton), Nova Scotia, showing location of 5 Mi’kmaq communities (Wagmatcook, Waycobah, Potlotek, Eskasoni, Membertou) participating in this study.

During the first 2 phases (face-to-face interviews with Mi’kmaq parents and children affected by asthma followed by an intervention in the form of an asthma camp), we identified the support resources, support-seeking strategies, support and education needs, and intervention preferences of Mi’kmaq children with asthma and their parents or caregivers (15). We found a lack of community support for affected Mi’kmaq families, despite a strong desire for these services. In this article we share final phase (Phase 3) findings. In this phase, we communicated the perspectives of Mi’kmaq parents and children to local health and education professionals and then elicited their perspectives to better understand the implications for health promotion programs, policies, and practices.

## Methods

A research partnership with the health directors of the 5 Mi’kmaq communities in Unama’ki (Tui’kn Partnership) was established; multiple planning meetings were held during Year 1; and 6 Mi’kmaq community researchers were hired to assist with participant recruitment, data collection, analysis, and knowledge sharing within the communities across all phases of the project (Year 2 and 3). A key factor in implementing the study was the community researchers’ ties to their own communities.

In Year 3, between July and September 2012, 22 health care professionals (ie, nurses, doctors, and community health representatives) and school board personnel (ie, teachers, coaches, and principals) were recruited from the 5 Mi’kmaq communities (Table 1). Participants were recruited on the basis of their involvement in Mi’kmaq education or community health programs, policy, or practices. Participants contributed to 1 of 5 focus groups (3–6 participants per focus group) held at each of the 5 community health centers. Both the Dalhousie Health Sciences Research Ethics Board and the Mi’kmaq Ethics Watch (a community-based ethics oversight and review board) approved the study protocols.

**Table 1:** Characteristics of Focus Group Participants, Mi’kmaq Communities, Unama’ki, Canada, 2012

Occupation	Mi’kmaq/Non-Mi’kmaq	Sex	No. of Participants
**Membertou Focus Group (July 25, 2012)**			
Health professional	Non-Mi’kmaq	Female	1
Health professional	Mi’kmaq	Female	2
Health paraprofessional	Mi’kmaq	Female	1
Health professional in training	Mi’kmaq	Female	1
**Total no. of participants**			**5**
**Waycobah Focus Group (August 16, 2012)**			
Health professional	Non-Mi’kmaq	Female	1
Health professional	Mi’kmaq	Female	3
School professional	Non-M’kmaq	Female	1
**Total no. of participants**			**5**
**Potlotek Focus Group (August 22, 2012)**			
Health professional	Non-Mi’kmaq	Male	1
Health professional	Non-Mi’kmaq	Female	1
Health professional	Mi’kmaq	Female	1
**Total no. of participants**			**3**
**Eskasoni Focus Group (September 7, 2012)**			
Health professional	Non-Mi’kmaq	Male	1
Health professional	Mi’kmaq	Female	2
School professional	Non-Mi'kmaq	Male	1
School professional	Mi'kmaq	Female	2
**Total no. of participants**			**6**
**Wagmatcook Focus Group (September 26, 2012)**			
Community health professional	Non-Mi’kmaq	Female	2
Community health professional	Non-Mi'kmaq	Male	1
**Total no. of participants**			**3**

Focus groups were facilitated by a non-Aboriginal research team member (R.W.), who served as the Nova Scotia site coordinator under the supervision of the site’s academic research lead (H.C.). Immediately before each focus group, participants were given 15 minutes to read a booklet that summarized the study’s purpose and Phase 1 and 2 results (http://www.heclab.com/wp-content/uploads/2015/01/Asthma-Booklet-Version-21-Real-Final-July-20.pdf); an oral account of the contents was also provided to ensure common understanding among participants. The facilitator used a semistructured interview guide containing open-ended questions that allowed participants to comment on Phase 1 and 2 results, their usefulness for planning and designing Mi’kmaq-specific asthma programs, recommendations for improved community support, and appropriate education strategies. Focus groups lasted approximately 1 hour each and were digitally recorded and transcribed. Supplementary field notes and observations by R.W. (with participants’ permission) were also included in the data to capture conversations that occurred when the audio recorder was turned off.

A thematic content analysis was undertaken, whereby text was assigned either latent (ie, interpretive or inferential) or manifest (ie, in vivo or actual words) codes (18). Once a list of codes was generated, they were sorted into themes. The final set of themes was then compared with themes reported in the literature, and the study objectives and the results were discussed with the 5 health directors (Tui’kn Partnership) to ensure credibility and transferability (19).

## Outcomes

Our analysis revealed 3 key areas of opportunity or challenge for implementing asthma prevention and management initiatives in Mi’kmaq communities: 1) professional awareness, 2) local school issues, and 3) community health centers. Each key area is described below. Representative participant quotes are provided (Table 2).

**Table 2:** Quotes From Focus Group Participants, Mi’kmaq Communities, Unama’ki, Canada, 2012

Theme	Quote ID	Quote
Lack of professional awareness about extent of asthma and asthma-related issues and needs	1	Asthma is not one of the more prevalent problems . . . .There are other[s] that seem to be more at the forefront with the population in this community. [nurse practitioner]
	2	In the 23 years I have been working at the school, I have seen very few kids with asthma that I am aware of. If [the students] don’t tell us [about their condition], we don’t really know . . . [this study] brings awareness that there may be some significant cases of asthma in our schools that we currently don’t know about. [Physical education teacher]
	3	The part of the study I find most useful is that [Mi’kmaq families] say they have no supports . . . This is interesting since we could . . . talk about [asthma] with the schools, I mean we just don’t think of that. I usually think of a support group as Alcoholics Anonymous or something like that. [Community health director]
	4	I see the study providing baseline information for moving forward with developing policy [within the school board], especially when people see that teachers are not letting the children have puffers [at school or in their possession] . . . . [T]he school system needs an eye opener into the disease itself and how it affects the children . . . . I see the study being influential in supporting this process. [Nurse practitioner]
School-based opportunities and challenges	5	What would be most beneficial is to have [some kind of intervention] set up in the schools. [The school staff] is the front line with the kids who are spending the majority of their time with these people. If we can educate [school personnel] on what they can do to create a safer environment [for asthmatic children], that would be most beneficial. [Licensed practical nurse]
	6	A lot of times these [asthmatic children] are being limited in the types of school activities they are allowed to participate in . . . [With improved teacher education], activities can be modified to accommodate the child’s specific needs so they can still participate. When they don’t get to participate, it’s social isolation. They become introverted. We want to encourage [asthmatic children] to grow and be successful members of the community but it is probably really difficult because they are socially isolated from their friends and limited in the activities they can take part in. [Licensed practical nurse]
	7	Between myself and [the other gym teacher] we teach 400 kids and by the time we get around to reading everyone’s file, it’s too late. [Physical education teacher]
Health center-based opportunities and challenges	8	It’s just difficult to get people to come to [support] groups. [Community members] say they want [support] groups but when you put them on they don’t attend. [Community health nurse]
	9	“[Community members] wanted to support [the work] of a community member rather than [health center staff]. [Community health nurse]
	10	There is interest [in another camp]. All those families who participated last summer asked me if there would be another asthma camp because they want [support]. I told them we’ll have to wait and see but there wasn’t. It’s too bad. [Community health nurse]
	11	We have limited amounts of knowledge on everything and not a lot of knowledge on anything. It would help if we had someone in-house that was your chest expert so they could be your “go to” person . . . . [Asthma] education for staff persons would be very relevant . . . [and] doable . . . . That would be a really good resource for the community and [asthmatic] children. [Nurse practitioner]
	12	There is a lot in [this study] about education. I can educate, but if I don’t know how the other side learns then I will not get my point across. [Non-Mi’kmaq doctor]

### Professional awareness about the extent of asthma and asthma-related issues and needs

The professionals who participated in Phase 3 indicated that Phase 1 and Phase 2 findings provided new information for asthma-related health programs and policy. Like the community members on the whole, many professionals had not considered asthma as a health priority even though it is one of the top 2 chronic illnesses affecting Aboriginal children (Table 2). Despite regular interaction with community children, professional participants could not recollect anyone openly self-identifying as having asthma, providing the (false) impression that asthma is not an issue in Aboriginal communities (Table 2).

In addition, several professional participants reported that, although they were aware of the challenges associated with asthma, they were unaware that families managing the condition required support. The strong demand from parents and caregivers for community health initiatives specific to asthma (15) revealed in Phases 1 and 2 surprised many professionals in Phase 3 (Table 2). Many health professionals would not see large numbers of visits or hospitalizations because there are no hospitals on-reserve; many families have to travel an hour or more to get to the nearest full-service emergency department. Moreover, many prefer to see health professionals off-reserve for greater anonymity about personal health care issues.

The reality that many schools had policies that put asthma sufferers at risk (eg, no prescription drugs allowed in schools or in the possession of students) was also identified as valuable, new information (Table 2, Quote 4). Professional participants indicated that becoming aware of the negative implications of this school policy provided them with the ammunition they needed to communicate with policy makers who could then initiate positive change in local schools.

Participants also identified several key audiences for education programs, specifically community leaders (ie, Mi’kmaq chiefs and councils), school administrators, funding agencies, and health directors). Distributing lay summaries of the findings, using social media (eg, Facebook), and establishing a community newsletter were also identified as effective in reaching the wider community. Several participants also suggested conducting additional research to determine specific asthma statistics for each community, to augment this study’s findings and provide more clout in terms of changing federal and local policy and increasing funding.

### School-based opportunities and challenges

Community health professionals and school staff identified opportunities for improving asthma support through local schools. School-based initiatives such as “lunch and learn” events, guest speakers, and regular seminars on asthma were reported as ideal ways to improve asthma support for Mi’kmaq children (Table 2). Health care professionals recognized the importance of school-based asthma education for improving not only the management of the physiological dimensions of asthma but also the psychosocial barriers some children with asthma encounter (Table 2).

Our Phase 1 and 2 results (15) indicated that Mi’kmaq children living with asthma relied on peer as well as parental support, which is consistent with asthma support strategies for children in general (20). Given this finding, Phase 3 professional participants recognized that, in addition to educating school staff, educating the entire student population was important for building awareness and support and because children tend to share what they learn at school with their family members at home.

Overall, Phase 3 professional participants offered several school-related recommendations. For example, physical education instructors reported that the high volume of student medical files made identifying students with asthma challenging (Table 2). They noted that much of the students’ information pertained to mental health issues and learning disabilities rather than chronic diseases, suggesting a potential area for improving reporting mechanisms. Reluctance among children to disclose their condition also exacerbated reporting issues, and participants agreed that children with mild to moderate symptoms often hesitated to openly identify as having asthma for fear of exclusion, isolation, and stigma.

### Health center-based opportunities and challenges

Phase 3 participants identified 3 key health center-based opportunities and challenges for improving community asthma support. First, health professionals and school personnel agreed that the health interventions (ie, support groups, asthma seminars, monthly asthma education nights, asthma camps) recommended by Mi’kmaq families in Phase 1 and 2 offered potential solutions to the community’s asthma-support deficiencies. However, an indication of how challenging it would be to provide such initiatives is the lack of participation in face-to-face support group sessions specific to other chronic conditions (Table 2). Numerous explanations were offered, including non-Aboriginal health care professionals’ lack of understanding of Aboriginal peoples’ lived experiences and these professionals’ lack of competence in culturally relevant communication protocols. All 5 focus groups identified keys (though not guarantees) to achieving moderate to strong success: 1) ensure that initiatives are advertised well in advance, and 2) provide transportation, childcare, food, personalized invitations, culturally relevant education, and hands-on activities.

Limitations to providing asthma support and promoting health centered on funding. Professional participants discussed other health-related issues that they and community members see as higher priorities than asthma (eg, diabetes, obesity, cancer, mental health, suicide). These conditions therefore compete with asthma for funding and face the same general difficulties in securing long-term funding. Professional participants reported that efforts to support chronic disease in Mi’kmaq communities are ineffective in reaching target audiences (21), whereas health initiatives spearheaded by community members have had more success. One group of participants referred to a community-member who organized a cancer support session that drew more participation than the health center had ever had (Table 2). Although community health professionals remain willing to set up asthma support initiatives on their own, they recognized that a community-based asthma champion might result in stronger uptake, and they suggested that they could funnel resources through that individual if the opportunity arose.

Second, before reading about the Phase 1 and 2 results in the pre-focus group booklet, professional participants were already aware of the success of the 2-day asthma camp in supporting participating Mi’kmaq families and improving asthma awareness in the community. By success, we mean that implementing an annual asthma camp was recognized as a viable asthma support opportunity, and health professionals in all 5 communities had fielded inquiries regarding the prospect of another camp — clear indicators of support and increased awareness about this chronic disease (Table 2). However, limited and competing financial priorities as well as limited human resource capacity to organize such events were barriers to operating an annual camp.

Third, opportunities for improved professional asthma support within the health centers were identified as important. During the asthma camp intervention, parents or caregivers noted that they were reluctant to seek outside expertise for 3 reasons: 1) they encountered racism in many health care settings; 2) there were complications and uncertainties associated with federal policies for Aboriginal health service and medication coverage; and 3) they often received different advice from different health care providers regarding prescriptions and symptom management. In response, Phase 3 participants recognized that the staff of the 5 health centers lacked asthma expertise. Participants proposed that investing in asthma training might reduce the need for families managing the condition to seek asthma expertise outside the community (Table 2).

Although providing asthma education was an underlying theme among study participants, several indicated that more effort was needed to understand how to effectively communicate across cultural and epistemological differences (Table 2). Participants indicated that cultural competence was essential to effectively address community-level asthma education gaps. That is, non-Mi’kmaq knowledge providers needed to understand the Mi’kmaq worldview of health as well as, and especially, the distal determinants of health (eg, colonialism and racism).

## Interpretation

Our findings provide new and relevant information for the development in Mi’kmaq communities of asthma programs, policy, and practices that are transferable to other Aboriginal communities. Many Aboriginal communities in Canada — and Indigenous peoples around the world with similar colonial histories (eg, New Zealand, Australia, United States) — share similar characteristics with respect to the physical and social conditions that affect asthma and asthma symptoms. At the same time, we caution against making sweeping generalizations when interpreting our results, given the epistemological and cultural diversity of individual communities.

Our earlier Phase 1 and 2 results (15), shared directly with community-based health and education professionals through Phase 3, have brought awareness of asthma as a pressing issue for families of children with asthma. Our results are positioned to reconcile existing misconceptions regarding the perceived lack of community asthma incidence and promote the implementation of relevant, community-wide asthma initiatives, applying a “social determinants of Aboriginal health” lens to health promotion principles.

Because of their limited encounters with self-reporting children, many health and educational professionals participating in this study were under the (misguided) impression that there were few cases of asthma in their communities and that it was not a pressing health concern. This lack of professional awareness and expertise is consistent with the nature of asthma in Aboriginal communities: it is often underdiagnosed, untreated, and hidden from those outside the immediate support network (usually, the family) (7). Until the challenges associated with identifying people with asthma (eg, no family physician, symptom-based diagnosis without spirometry testing) are overcome, lack of community and professional awareness will probably remain.

Our findings reinforce the need for improved school policy and practices. School boards need to create asthma-friendly policies that give students immediate access to their medication, thereby encouraging children to take control of their condition, which is an integral part of effective chronic disease management (22). Making certain school-based activities more inclusive for children with asthma could reduce the social isolation they experience; for example, modifying the degree of physical exertion necessary to participate in gym class to suit individuals. Modifying physical activities is only a short-term and inadequate solution until the asthma therapies for these children are better controlled (ie, the upstream determinants of health as well as the gaps in health services are addressed in Mi’kmaq and other Aboriginal communities). In addition, modifying activities is contrary to many professional guidelines available for managing asthma (eg, the American Clinical Practice’s Stepwise approach). But the unique circumstances of on-reserve health care require that adjustments be made to avoid the unintended consequences of waiting until professional guidelines can be fully implemented. Providing asthma education for students and school staff will increase awareness of both the condition and the implications that school policy has on children with asthma. Given the general reluctance of students with asthma to disclose their condition, improved communication between teachers and students and streamlining the process through which teachers review students’ medical files and have individualized action plans (eg, moving from paper files to searchable electronic communications) are keys to mitigating acute events.

Similar to other chronic disease scenarios, effective asthma support should be provided in a way that is culturally meaningful (23). Our results, however, reveal a gap in asthma awareness and expertise across the 5 community health centers. Providing professional development in asthma support, education, and intervention for health center staff, many of whom are Mi’kmaq, will mean that community members seeking advice from an asthma specialist have an accessible (and culturally and linguistically compatible) community resource at their disposal, reducing existing social (ie, racism) and physical (ie, geographic distance) barriers to obtaining support (15). However, with multiple competing health priorities stemming from the gross social, economic, and health disparities that pervade Aboriginal communities — and with asthma being largely absent from public discourse — any community’s leadership will probably face barriers to adequately addressing the issue or accessing the financial resources to do so.

Our study finds that asthma support initiatives in schools and community health centers, and through culturally appropriate asthma camps, are promising community-driven ways of reducing the barriers facing Mi’kmaq families affected by asthma. Given the financial commitment required to initiate health interventions (our budget was $25,000 CAD), however, sustained support is needed from those responsible for allocating on-reserve funds and suggests the need for community health centers and schools to partner with other health service organizations. Engaging in educational strategies that are culturally meaningful may also help position asthma as an ongoing and pressing community concern in Mi’kma’ki and beyond.
